# Congenital Chagas disease: an update

**DOI:** 10.1590/0074-02760140405

**Published:** 2015-05

**Authors:** Yves Carlier, Sergio Sosa-Estani, Alejandro O Luquetti, Pierre Buekens

**Affiliations:** 1 Laboratoire de Parasitologie, Faculté de Médecine, Université Libre de Bruxelles, Bruxelles, Belgique; 2 School of Public Health and Tropical Medicine, Tulane University, New Orleans, LA, USA; 3 Instituto Nacional de Parasitología Dr Mario Fatala Chaben, Ministry of Health, Buenos Aires, Argentina; 4 Hospital das Clínicas, Universidade Federal de Goiás, Goiânia, GO, Brasil

**Keywords:** Trypanosoma cruzi, congenital *T. cruzi* infection, congenital Chagas disease, maternal-foetal transmission

## Abstract

Congenital infection with Trypanosoma cruzi is a global problem, occurring on average in 5% of children born from chronically infected mothers in endemic areas, with variations depending on the region. This presentation aims to focus on and update epidemiological data, research methods, involved factors, control strategy and possible prevention of congenital infection with T. cruzi. Considering that etiological treatment of the child is always effective if performed before one year of age, the diagnosis of infection in pregnant women and their newborns has to become the standard of care and integrated into the surveillance programs of syphilis and human immunodeficiency virus. In addition to the standard tests, polymerase chain reaction performed on blood of neonates of infected mothers one month after birth might improve the diagnosis of congenital infection. Recent data bring out that its transmission can be prevented through treatment of infected women before they become pregnant. The role of parasite genotypes and host genetic factors in parasite transmission and development of infection in foetuses/neonates has to be more investigated in order to better estimate the risk factors and impact on health of congenital infection with T. cruzi.

At least two million women in fertile age are estimated to be chronically infected with *Trypanosoma cruzi* in Latin America with the incidence of congenital infection being at least 15,000 cases/year (OMS/OPS 2006). Maternal-foetal transmission may be repeated in each pregnancy (family clustering of congenital cases) and can occur from one generation to another (vertical transmission) [reviewed in Carlier and Truyens (2010)].

Since congenital infection with* T. cruzi* (i) is currently mostly silent (asymptomatic), (ii) may progress to severe chronic Chagas disease later in life and (iii) can be effectively cured if treated [with benznidazol (BZ) or nifurtimox (NFX)] within the first year of life, its diagnosis is of upmost importance. A consensus has been established on the control strategy to be applied and gold standards for its laboratory diagnosis have been recommended (Carlier et al. 2011).

The testing process begins by identifying pregnant women living or who have lived in endemic areas or having received blood transfusion or organ transplants in endemic areas or who were born to an infected mother, assuring they are really infected. *T. cruzi*-specific antibodies are identified using two serological tests. Conventional tests, such as ELISA, indirect immunofluorescence and indirect haemagglutination, whose performance depends on good quality testing kits and good laboratory practice, have been in use since 1975 in all endemic countries. Rapid tests can be used but need to be confirmed by standard serological tests. Tests can be performed during pregnancy (prenatal) or on umbilical cord blood (containing transferred maternal IgG antibodies) (Sosa-Estani et al. 2008). If the test results are negative, there is no possible transmission.

After confirmation of infection in the mother, parasites have to be sought in the newborn from close-to-birth up to one month after birth using parasitological tests (e.g. micro-haematocrit, haemoculture) if resources exist in the health systems and/or by serological methods after eight months of age when maternal transferred IgG antibodies have disappeared. If parasites are observed (microscopic examination) or specific antibodies are detected after eight months, infection is proved and the infant should be treated (Fig. 1).

**Fig. 1:**
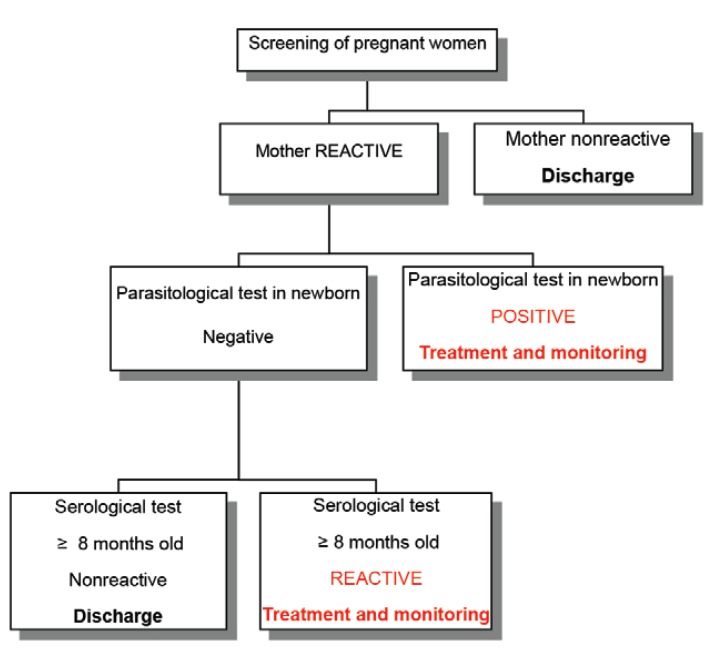
decision algorithm in the control strategy of congenital infection with Trypanosoma cruzi.

This presentation aims to focus on and update epidemiological data and research methods on congenital infection with *T. cruzi*, the factors involved in parasite transmission and development of infection in foetuses/neonates and its control strategy and its possible prevention.

Present situation of congenital infection with T. cruzi/congenital Chagas disease in endemic and non-endemic countries.

Although both terms are often confused, "congenital infection with *T. cruzi*" refers to asymptomatic as well as symptomatic cases of infection, whereas "congenital Chagas disease" is mainly used to describe symptomatic cases. The maternal-foetal transmission rate, classically defined as the number of congenital cases/number of *T. cruzi*-infected mothers (Carlier & Truyens 2010), can gain in precision if expressed as the number of congenitally infected infants divided by the number of infants born to infected mothers (including multiple births) (Howard et al. 2014).

Although maternal-foetal transmission of *T. cruzi* occurs in an average of 5% of chronically infected mothers (the most frequent clinical form of Chagas disease) in endemic areas, major differences seem to occur among the different countries. The largest surveys have been performed in endemic countries located in the South Cone of Latin America. For example, congenital *T. cruzi* infection was detected in 431 of 7,188 children in Argentina (6%), in 292 of 7,086 in Bolivia (4.1%) and in 115 of 2,691 in Paraguay (4.3%) (Howard et al. 2014). Studies from Brazil and other endemic countries include shorter series. Nevertheless, in a survey performed from 2004-2012 in the state of Goiás (GO) in Brazil 910 of 1,773 pregnant women (51.3%) were confirmed as seropositive for *T. cruzi *and only eight of 894 (0.9%) delivered an infected newborn (AO Luquetti, unpublished observations). In a larger seroepidemiological study (performed from 2001-2008) of 104,813 children under five years old and living in vector-free areas (detected infection is here considered to be congenital) of all Brazil, only 20 (as well as their mothers) were infected (0.02%), 12 of them coming from a single state (Rio Grande do Sul), bordering Argentina and Paraguay (Table) (Luquetti et al. 2011). A recent systematic review of Brazilian data from 1984-2009 indicates a pooled congenital transmission rate of 1.7% (Martins-Melo et al. 2014), i.e., less than in the American South Cone countries.

**TABLE:** Distribution of children congenitally infected with Trypanosoma cruzi by Brazilian statea

State	Samples (n)	Cases^*b*^ (n)	Proportion^*c*^ (%)
Alagoas	3,742	1	0.03
Bahia	16,577	2	0.01
Minas Gerais	11,386	3	0.03
Paraná	3,424	1	0.03
Pernambuco	7,140	1	0.01
Rio Grande do Sul	4,569	12	0.26

a : data from the national survey of seroprevalence, Brazil 2001-2008 (Luquetti et al. 2011);

b : number of confirmed congenital cases after venous collection of blood of the child and the respective mother by four different serological tests;

c : proportion of the final number of confirmed cases of congenital transmission (areas free of vector transmission) among the number of samples collected in filter paper from children below five years of age.

Congenital transmission has also been recorded from non-endemic countries as a consequence of migration of infected pregnant women from Latin America (mainly Bolivia), in the United States of America, Spain, Switzerland and Sweden. From the 18 studies performed in Spain, 32 children from the 743 born to infected mothers were found infected (4.3%) and in another study from Switzerland two children were infected from eight [reviewed in Howard et al. (2014)].

Whether the local variations in the distribution of congenital infection with *T. cruzi* are due to the different methods used to detect it or to known or still unknown factors involved in transmission and/or development of congenital Chagas disease (see below), well-framed large population-based studies are needed to better measure its frequency, risk factors and impact on health.

Epidemiologic research methods for field studies of congenital T. cruzi infection/congenital Chagas disease

Different epidemiologic designs can be used to study congenital *T. cruzi* infection at the population level. Approaches include (i) prospective cohort studies, measuring maternal antibodies during prenatal care or on umbilical cord blood and following-up the infants (Buekens et al. 2013), (ii) case-control studies comparing cases with rare outcomes to healthy controls and retrospectively assessing if their mothers were *T. cruzi* infected and (iii) cross-sectional studies, which are often household surveys (Gamboa-León et al. 2014). Point-of-care rapid tests and tests performed on dried blood spots on filter papers facilitate the serological testing in the field.

In cohort studies, challenges to identify *T. cruzi* infected infants include having trained personnel available 24/7 to perform direct parasitological examination of cord blood and of infants' blood obtained by heel prick within a few hours after blood collection, and organising follow-up household visits to perform *T. cruzi* serology after the disappearance of maternal antibodies after eight months of age. Mobile Health approaches to contact mothers by cell phone and geographic information systems are helpful to facilitate household visits.

Factors involved in maternal-foetal transmission of T. cruzi infection and development of congenital Chagas disease

Four main factors (parasite, mother, placenta, foetus) are involved in the transmission and/or the development of congenital Chagas disease. Since the transmission of parasites through breast feeding (postnatal), amniotic fluid or by transuterine route are unlikely (Virreira et al. 2006 b, Carlier & Truyens 2010, Norman & López-Vélez 2013), the haematogenous transplacental route is the only possibility for prenatal or perinatal transmission.

*Role of parasite* - The capacity of parasites to invade placental cells (see below), as well as their virulence and amount in pregnant women (particularly in retroplacental blood), is doubtless involved in their maternal-foetal transmission, as likely are their virulence and amount in the development of congenital Chagas disease in foetuses/neonates. It remains to be determined if such features depend on parasite genotypes as defined today. Indeed, various genotypes of *T. cruzi *(TcI, TcII, TcIII, TcV and TcVI) and associations of different genotypes have been identified in cases of congenital infection. The genotype TcV has been reported in 80-100% of congenital cases in Argentina, Bolivia, southern Brazil, Chile and Paraguay, whereas TcII has been identified in the few cases detected in the other Brazilian states (Virreira et al. 2006a, 2007, Burgos et al. 2007, Corrales et al. 2009, Garcia et al. 2014, AO Luquetti et al., unpublished observations); so, whether e.g. TcI, TcII or other genotypes are transmitted less frequently and involved less in the development of congenital infection remains to be investigated.

*Role of parasitaemia and immune status of pregnant women* - Parasitaemia increases during pregnancy (2nd and 3rd trimesters) and high maternal parasitaemias are associated with congenital transmission (Hermann et al. 2004 , Virreira et al. 2007, Brutus et al. 2010, Siriano et al. 2011, Bua et al. 2012 ). Indeed, transmission occurs in nearly 100% of pregnant women with reactivated infections [as, e.g., in case of co-infection with human immunodeficiency virus (HIV)] (Scapellato et al. 2009), in 53% of acute infection during pregnancy (8/15 of reported cases) (Brabin 1992, Moretti et al. 2005) and in roughly 5% of chronic infection in endemic countries, displaying huge, high and hardly detectable parasitaemias, respectively (Fig. 2).

**Fig. 2:**
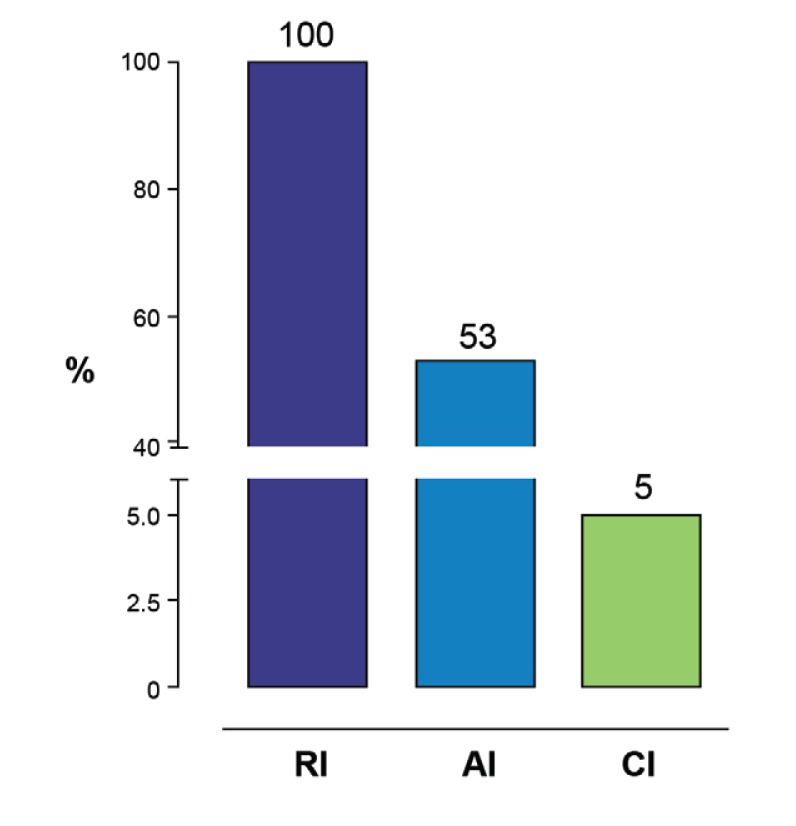
maternal-foetal transmission rates in acute (AI), chronic (CI) and reactivated (RI) Trypanosoma cruzi infection.

Maternal-foetal transmission of parasites is also associated with some degree of maternal immune deficiency: a lower innate immune response with reduced leukocyte release of tumour necrosis factor (TNF)-α (García et al. 2008) and a lower T-cell-mediated specific type 1-immune response with reduced production of interferon (IFN)-γ (Hermann et al. 2004), that likely contribute to increased maternal parasitaemia.

Why can transmission be repeated in each pregnancy or be transgenerational (see above)? Are there predisposed mothers with a reduced capacity of T-cell-mediated response? These unanswered questions should encourage studies on the possible role for host genetic factors favouring parasite transmission.

*Role of placental defences and route of transmission* - The presence of parasites in intervillous blood induces the release by placenta of proinflammatory cytokines, chemokines, as well as reactive oxygen and nitrogen intermediates that are susceptible to kill parasites (Díaz-Luján et al. 2012). However, in vitro studies show that *T. cruzi* can infect and multiply within human villous trophoblastic cells (Shippey et al. 2005, Castillo et al. 2013).

What are the lessons of the histopathologic studies of placentas from infected foetuses/neonates? Roughly, two situations have been observed: (i) in severe and mortal congenital Chagas disease (rare today, but frequent in the past), a severe placentitis/villitis occurs with destruction and rupture of the trophoblastic barrier; parasites are found in villous trophoblast and stromal cells, cytokines, such as TNF-α, are intensively produced and, in such cases, the placental innate defences are likely overflowed by huge parasitaemias in the intervillous space (it is probably what occurs also in experiments in vitro); the rupture of the trophoblastic barrier further increases foetal infection and TNF-α contributes to abortion and neonatal mortality (Bittencourt 1988, Altemani et al. 2000); (ii) in mild congenital *T. cruzi* infection in live neonates (frequent today), villitis is much less marked or not observed, parasites are not or hardly identified in villous trophoblast (Moya et al. 1979, Azogue et al. 1985, Altemani et al. 2000, Fernandez-Aguilar et al. 2005, Carlier & Truyens 2010) and this indicates that trophoblast remains a barrier for *T. cruzi*, and haematogenous transmission to foetuses has to take alternative transplacental routes.

What are the possible alternative transplacental routes? Two can be identified: (i) the placental marginal zone (joining the membranes to the chorionic and basal plates, constituted of smooth muscle cells embedded in an extracellular matrix, only covered by a nontrophoblastic epithelium); serial biopsies performed in 19 placentas from infected live Bolivian newborns showed the densities of parasites were particularly high and gradually decreased in the chorionic plate and distant membranes inducing chorionitis/chorioamnionitis; (ii) the placental breaches/tears appearing naturally close to the pregnancy term (labour contraction-mediated damages) (Fernandez-Aguilar et al. 2005, Carlier & Truyens 2010).

Parasites enter the chorion and, surviving the mesenchymal second placental line of defence, spread by successive infections of fibroblasts and macrophages to finally gain access to foetal vessels embedded in chorionic plate and umbilical cord.

*Parasitaemia and immune status of foetuses/neonates* - Morbidity and mortality of congenital Chagas disease are associated with high parasitaemias, i.e., rely on the capacity of transmitted parasite to multiply into the foetus/neonate (virulence). Asymptomatic congenital cases generally display at birth less than 100 p/mL in blood while 100-1,000 p/mL can be found in symptomatic cases and the rare lethal clinical forms can reach up to 125,000 p/mL (Torrico et al. 2005, Virreira et al. 2007, Bua et al. 2012).

This raises the question of the capacity of foetuses/neonates to control infection despite their alleged immunological immaturity. Both innate and adaptive immune responses have been observed in foetuses/neonates. Inflammatory responses are induced in uninfected neonates born to *T. cruzi*-infected mothers (Vekemans et al. 2000, García et al. 2008, Cuna et al. 2009) and neonatal dendritic cells (Rodriguez et al. 2012), natural killer cells (Guilmot et al. 2014 ) and monocytes (Guilmot et al. 2013) can be activated by *T. cruzi. *The capacity of activated monocytes to eliminate parasites opsonised by transferred maternal antibodies might protect offspring of infected mothers against the development of congenital infection (capacity of self-cure?) (Truyens & Carlier 2010). Moreover, infected foetuses/neonates display activated and cytotoxic CD8 T cells that produce IFN-g in response to parasites (Hermann et al. 2002). However, such specific capacity to produce IFN-g is drastically reduced in neonates displaying high parasitaemias, i.e., in severe and lethal forms of congenital Chagas disease (Torrico et al. 2005), indicating that immune defences are either lacking, insufficient or initiated too late in congenitally infected neonates and become unable to control multiplication of parasites transferred from their mother. Altogether these data strongly suggest that most current congenital infections with *T. cruzi* result from: (i) weak maternal innate and adaptive type 1 immune responses enhancing parasite multiplication and high parasitic loads in retroplacental blood, (ii) an haematogenous route of parasite transmission through placental areas deprived of trophoblast and (iii) insufficient foetal/neonatal innate defences and parasite-specific type-1 immune response to control multiplication of transmitted parasites. Therefore, whether parasites in maternal blood can be either not transmitted (there is no congenital infection in case of sufficient maternal immune responses) or transmitted to the foetus with the subsequent development of a congenital Chagas disease (in case of weak foetal/neonatal immune response), the possibility of transmission followed by a rapid elimination of parasites by the foetal immune responses cannot be excluded (in this case of self-cure, there would be a brief congenital infection without development of congenital Chagas disease). More investigations are needed to specify the role of parasite genotypes in such mechanisms and of host genetic factors governing the intensity of immune responses towards parasites.

## Prevention and control of congenital T. cruzi infection/congenital Chagas disease

The control strategy of congenital infection defined above (Carlier et al. 2011) can be improved by analysing why opportunities for its proper diagnosis are lost in health systems.

*Analysis of losses of opportunities of adequate diagnosis of congenital infection* - In the health system, such losses can be due to: (i) low attendance to prenatal care for diagnosing infection in pregnant women (low priority for health systems), (ii) low rate of infection detection by parasitological tests or (iii) health workers do not know the procedures, do not have adequate training and do not have good working conditions. At the community level, mothers do not know the procedure and/or do not understand the instructions and/or do not have financial resources to travel to the health centres.

*Potential solutions in health systems and communities* - *T. cruzi* serology has to be integrated in national or local programs of prenatal screening with syphilis and HIV. Such surveillance has to become the standard of care. If prenatal screening is not possible, serological diagnosis has to be performed at delivery. Information, education and communication programs on Chagas disease and its congenital transmission have to be strengthened at the community level. Some successful programs in two states of Brazil (Mato Grosso do Sul and GO) are currently underway (Botelho et al. 2008, Siriano et al. 2011).

*Tests to improve timely laboratory diagnosis of congenital infection* - More experience has been accumulated using polymerase chain reaction (PCR) for diagnosis of congenital *T. cruzi* infection. In addition to its known higher sensitivity over parasitological methods when used at birth (Virreira et al. 2003, Mora et al. 2005), PCR performed repeatedly at different time points after birth has been shown to allow an earlier diagnosis of congenital infection [positive PCR are mainly obtained 1.5 months after birth, whereas the micro-haematocrit tests are positive at an average of 6.7 months and serology at 11.8 months (Velázquez et al. 2014)]. PCR detects more infected children at 30 days after birth, when parasite burden is at its maximum (Bua et al. 2012, 2013). Moreover, using PCR one month after birth rather than at birth might reduce the risk of false positive diagnosis in case of transmission of parasite DNA debris from mother or self-cure by the foetus/neonate (see above).

Detection of antibodies against shed acute-phase antigen (SAPA) in maternal and offspring blood seems promising to identify congenital *T. cruzi* infection within 30 days after birth, when the mother is SAPA nonreactive and the child is SAPA reactive or when the differential of SAPA using matched samples of mother and child is ≥ 1. In contrast, the offspring does not have congenital *T. cruzi* infection when the child is SAPA nonreactive or both samples of the mother and child are reactive, but with a differential < 1 (Mallimaci et al. 2010, Russomando 2010). However, experience using detection of SAPA antibodies for the diagnosis of congenital *T. cruzi* infection remains limited to Paraguay and Argentina and would need the availability of reagents and to be extended to other areas before being recommended.

*Prevention of congenital infection is possible* - Recent results have shown the benefit of trypanocidal treatment in girls or nonpregnant women to prevent congenital transmission to their newborns (Sosa-Estani et al. 2009, Fabbro et al. 2014). Three hundred fifty-four nonpregnant women living in urban areas of Argentina and infected with *T. cruzi* were followed during 16 ± eight months: 132 were treated with BZ (5 mg/kg/day) or NFX (10 mg/kg/day) for 30-60 days and 222 had not received treatment. Children born to these mothers were investigated for occurrence of congenital transmission of *T. cruzi* by standard methods. Analysis of results indicated that children of untreated women (34 infected out of 222) have 21 times more risk of congenital infection with *T. cruzi* than children born to women treated before pregnancy (0 infected out of 132), indicating that the etiological treatment of infection before pregnancy is an effective strategy for primary prevention of congenital *T. cruzi* infection/Chagas disease.

## Concluding remarks

Congenital transmission of *T. cruzi* is a global problem, occurring on average in 5% of children born from chronically infected mothers in endemic areas, with variations depending on the region. Since the diagnosis tools used can at least partially explain such variations, larger population-based studies using similar methods are needed to better measure the frequency of congenital *T. cruzi* infection/Chagas disease. A better knowledge of the factors involved in its transmission and development and particularly the role of parasite genotypes and host genetic factors, should allow a better estimation of its risk factors and impact on health outcomes. Considering that etiological treatment of the child is always effective if performed before one year of age, the diagnosis of infection in pregnant women and their newborns is mandatory. In addition to the standard tests, PCR performed on blood of neonates of infected mothers one month after birth might improve such diagnosis. The availability of more simple and accurate diagnostic methods easily implemented in primary health care settings could provide tools for the timely treatment of infected newborns. Additionally, evidences are showing that congenital transmission could be prevented through treatment of infected women before they become pregnant.
